# Antigen Presenting Cells Link the Female Genital Tract Microbiome to Mucosal Inflammation, With Hormonal Contraception as an Additional Modulator of Inflammatory Signatures

**DOI:** 10.3389/fcimb.2021.733619

**Published:** 2021-09-16

**Authors:** Elizabeth H. Byrne, Mara Farcasanu, Seth M. Bloom, Nondumiso Xulu, Jiawu Xu, Barry L. Hykes, Nomfuneko A. Mafunda, Matthew R. Hayward, Mary Dong, Krista L. Dong, Thandeka Gumbi, Fransisca Xolisile Ceasar, Nasreen Ismail, Thumbi Ndung’u, Christina Gosmann, Musie S. Ghebremichael, Scott A. Handley, Caroline M. Mitchell, Alexandra-Chloé Villani, Douglas S. Kwon

**Affiliations:** ^1^Ragon Institute of Massachusetts General Hospital (MGH), Massachusetts Institute of Technology (MIT), and Harvard, Cambridge, MA, United States; ^2^Department of Medicine, Harvard Medical School, Boston, MA, United States; ^3^Division of Infectious Diseases, Massachusetts General Hospital, Boston, MA, United States; ^4^HIV Pathogenesis Programme (HPP), The Doris Duke Medical Research Institute, University of KwaZulu-Natal, Durban, South Africa; ^5^Department of Pathology and Immunology, Washington University School of Medicine, St. Louis, MO, United States; ^6^Females Rising Through Education, Support, and Health (FRESH), Durban, South Africa; ^7^Health Systems Trust, Durban, South Africa; ^8^Africa Health Research Institute (AHRI), Durban, South Africa; ^9^Max Planck Institute for Infection Biology, Berlin, Germany; ^10^Division of Infection and Immunity, University College London, London, United Kingdom; ^11^Department of Obstetrics and Gynecology, Massachusetts General Hospital, Boston, MA, United States; ^12^Center for Immunology and Inflammatory Diseases, Department of Medicine, Massachusetts General Hospital, Boston, MA, United States; ^13^Broad Institute of MIT and Harvard, Immunology Program, Cambridge, MA, United States

**Keywords:** HIV, female genital tract, microbiome, inflammation, mucosal immunology, hormonal contraception, host-microbiome interaction

## Abstract

The microbiome of the female genital tract (FGT) is closely linked to reproductive health outcomes. Diverse, anaerobe-dominated communities with low *Lactobacillus* abundance are associated with a number of adverse reproductive outcomes, such as preterm birth, cervical dysplasia, and sexually transmitted infections (STIs), including HIV. Vaginal dysbiosis is associated with local mucosal inflammation, which likely serves as a biological mediator of poor reproductive outcomes. Yet the precise mechanisms of this FGT inflammation remain unclear. Studies in humans have been complicated by confounding demographic, behavioral, and clinical variables. Specifically, hormonal contraception is associated both with changes in the vaginal microbiome and with mucosal inflammation. In this study, we examined the transcriptional landscape of cervical cell populations in a cohort of South African women with differing vaginal microbial community types. We also investigate effects of reproductive hormones on the transcriptional profiles of cervical cells, focusing on the contraceptive depot medroxyprogesterone acetate (DMPA), the most common form of contraception in sub-Saharan Africa. We found that antigen presenting cells (APCs) are key mediators of microbiome associated FGT inflammation. We also found that DMPA is associated with significant transcriptional changes across multiple cell lineages, with some shared and some distinct pathways compared to the inflammatory signature seen with dysbiosis. These results highlight the importance of an integrated, systems-level approach to understanding host-microbe interactions, with an appreciation for important variables, such as reproductive hormones, in the complex system of the FGT mucosa.

## Introduction

The microbiome of the female genital tract (FGT) has been implicated in a range of reproductive health outcomes. We have previously defined four discrete FGT microbial communities, termed cervicotypes (CTs), in South African women of reproductive age (Anahtar et al., 2015; Gosmann et al., 2017). Communities dominated by *Lactobacillus* species have been associated with favorable outcomes, while those with diverse anaerobes and low *Lactobacillus* abundance are associated with negative sequelae. Bacterial vaginosis (BV) is a clinical diagnosis in which these diverse communities are accompanied by vaginal discharge and associated symptoms. BV impacts up to 58% of women globally and is associated with a higher risk of sexually transmitted infections (STIs), including gonorrhea, chlamydia, herpes simplex virus type 2, and HIV [reviewed in (Brotman, 2011; Anahtar et al., 2018)], as well as preterm labor (Hillier et al., 1995; Meis et al., 1995; Anahtar et al., 2018). In sub-Saharan Africa, where HIV incidence remains high, particularly among young women, these dysbiotic vaginal communities are significantly more common than in the United States (Anahtar et al., 2015; UNAIDS, 2019). High prevalence of dysbiotic FGT communities has also been observed in other low and middle-income countries (Marconi et al., 2020), where women continue to face a disproportionate burden of global reproductive health challenges (Smid et al., 2016; Hull et al., 2020). The precise mechanistic links between the FGT microbiome and mucosal inflammation, the presumed link to these poor clinical outcomes, remain unclear.

Women’s health around the world, particularly in areas with limited access to healthcare, has been significantly improved through effective and accessible contraception. Due in part to its efficacy, low cost, and discrete nature, depot medroxyprogesterone acetate (DMPA) has remained a popular contraceptive option. In South Africa, more than a quarter of women use DMPA, accounting for nearly half of all contraception use in the country (Tsui et al., 2017; United Nations, 2020). DMPA has been suggested to modulate both the FGT microbiome and immune environment, complicating analyses of host-microbiome interactions in this setting (van de Wijgert et al., 2013; Roxby et al., 2016; Wessels et al., 2019; Noel-Romas et al., 2020). Progestin-only contraception, including DMPA, is associated with a decreased risk of BV (Vodstrcil et al., 2013) but with an increased FGT inflammatory state (Chandra et al., 2013; Morrison et al., 2014; Deese et al., 2015; Byrne et al., 2016; Morrison et al., 2018; Edfeldt et al., 2020). Although reproductive hormones and the microbiome are both known to modulate the FGT immune environment, it remains unclear how these paths might converge on genital inflammation.

In this study, we characterize transcriptional programs of specific cellular lineages in the endocervix that shed light on the host pathways contributing to microbiome-induced inflammation at this key mucosal barrier. We identify antigen presenting cells (APCs) as an important sensor of vaginal microbiota. We additionally address the effects of hormonal contraception on FGT inflammation and provide evidence that DMPA induces significant host transcriptional changes in multiple cell lineages. These results demonstrate that dysbiosis and DMPA modulate the mucosal environment of the FGT in distinct ways, highlighting the importance of using an integrated approach to more fully understand host-microbe interactions in the context of human clinical and behavioral covariates.

## Methods

### Sample Collection

Study specimens were obtained from participants in the Females Rising through Education, Support, and Health (FRESH) cohort, a prospective observational cohort study that enrolls 18- to 23-year-old, HIV-uninfected, non-pregnant women in Umlazi, South Africa. The cohort and its inclusion and exclusion criteria have been previously described in detail (Anahtar et al., 2015; Gosmann et al., 2017; Dong et al., 2018). The study protocol was approved by the Biomedical Research Ethics Committee of the University of KwaZulu-Natal (UKZN; Ethics Reference Number BF131/11) and the Massachusetts General Hospital Institutional Review Board (2012P001812/MGH). Participants provided informed consent.

All genital tract sampling was performed at the FRESH clinical research site in an exam room dedicated to pelvic examination. Sampling was performed by the same two trained research nurses throughout the study. Sampling was performed outside the menstrual period. Sample collection was performed under direct visualization by speculum exam. Lubricants were not introduced into the vagina prior to or during the speculum exam. The plastic single-use speculum was wet with tap water prior to insertion. Upon speculum insertion, swab samples (Puritan 6” Sterile Standard Foam Swab 126 w/Polystyrene Handle) were collected from the mid-vaginal wall, then from the ectocervical mucosa. A cervicovaginal lavage with 5 mL of sterile 0.9% saline (Adcock Ingram) was then performed, followed by collection of a cellular cytobrush (CooperSurgical, Medscand Cytobrush Plus, Catalog #C0005), which was rotated 360° in the cervical os. Cytobrushes were put into a 15 mL conical tube containing 5 mL of RPMI-1640 media supplemented with 10% v/v heat-inactivated fetal calf serum, 1% v/v Penicillin-Streptomycin-Amphotericin B 10K/10K/25ug mixture (Lonza Bioscience), L-glutamine (Lonza Bioscience, 2 mM final concentration), and HEPES (Lonza Bioscience, 10 mM final concentration). The tube was immediately placed on ice, and transferred to the processing lab at the HIV Pathogenesis Programme (HPP), Doris Duke Medical Research Institute, UKZN, for further processing (see details below). One cervical swab was used to make a microscope slide preparation for Gram stain analysis. The remaining cervical and vaginal swabs were placed in individual sterile vials on ice. A cervical swab was transferred, along with the prepared slide, to Neuberg Global Laboratories, Durban, South Africa, an accredited commercial laboratory diagnostics company, where polymerase chain reaction (PCR) testing was performed from the cervical swab for the sexually transmitted infections (STIs) *Neisseria gonorrheae*, *Chlamydia trachomatis*, *Trichomonas vaginalis*, and *Mycoplasma genitalium*. BV status was determined from the prepared slide by trained laboratory technologists at Neuberg Global Laboratories using the Gram-stain-based Nugent scoring method (Nugent et al., 1991). The remaining cervical and vaginal swabs were transferred to the HPP processing lab and stored at -80°C for subsequent sequence-based microbiome analysis.

Samples for host transcriptomic analysis were selected from participants who were STI-negative (chlamydia, gonorrhea, trichomonas, mycoplasma) at the time of sampling and who represented a distribution across the four CTs (**Table 1** and **Appendix 2**). Participants and samples were also selected based on maximal number of sorted cervical cells count, yielding comparable cell counts for each cell type across participants.

**Table 1:** Demographics of FRESH participants included in analysis.

Characteristic	CT 1/2 (n=13)	CT 3/4	p-value
Days since last sex (median, IQR)	18 [3, 32]	17 [6, 43]	0.600
Number of sexual encounters, last 30d (median, IQR)	2 [0, 2]	1 [0, 3]	0.762
Number of sex partners, last 30d (median, IQR)	1 [0, 1]	1 [0, 1]	0.581
Days since LMP (median, IQR)	45 [21, 315]	16 [1, 21]	0.009
Condom use during sex			0.550
Always	2	1	
Sometimes	4	7	
Never	3	2	
Drying agent use			0.613
Never	12	14	
Sometimes	1	3	
Contraceptive use			0.061
DMPA	4	4	
Nuristerate	3	0	
Implanon	1	0	
Lipez Loop	0	1	
None	5	12	

All women were enrolled between the ages of 18-23 years and all participants who were included in this analysis were HIV negative at the time of sampling. Data are presented as median [IQR] or number of participants, and p-values are calculated by Wilcoxon or Fisher’s Exact test. One participant was excluded due to samples failing to meet quality metrics during RNA sequence data processing (see Methods).

At the time of sampling, participants were asked about their current family planning methods and their last menstrual period (LMP). Women using no hormonal contraceptive were determined to be in the follicular phase of the menstrual cycle based on a self-reported LMP less than 14 days prior to sampling.

### Microbiome Sequencing and Analysis

Total nucleic acids from cervical or vaginal swab samples were extracted with a phenol-chloroform method, which included a bead beating process to disrupt bacteria as previously described (Anahtar et al., 2015; Anahtar et al., 2016). The V4 region of the bacterial 16S rRNA gene was PCR-amplified following standard protocols (Caporaso et al., 2012; Anahtar et al., 2015; Hoang et al., 2020). Amplicons were pooled, purified, and prepared according to standard Illumina protocols, and single-end sequenced on an Illumina MiSeq using a v2 300-cycle sequencing kit with addition of custom Earth Microbiome Project sequencing primers (Caporaso et al., 2012).

Sequence demultiplexing was performed as previously described (Hoang et al., 2020). Demultiplexed sequences were then processed using dada2 version 1.6.0 (Callahan et al., 2016) with taxonomy assignments performed using the RDP training database supplemented by manual curation (Anahtar et al., 2015). Tables with amplicon sequence variant (ASV) taxonomy, per-sample ASV read counts, species read counts, and sequencing metadata are detailed in **Supplementary File 1**. The denoised dada2 results with final taxonomic assignment were analyzed in R using phyloseq version 1.30.0 (McMurdie and Holmes, 2013) and custom R scripts (available in **Supplementary File 2**.

Sample read counts after dada2 processing all exceeded 13,000 reads per sample. For additional analysis, we excluded ASVs that could not be defined at least to the taxonomic level of class, that were not represented by >10 reads in >2 different samples, or that had an abundance of <50 reads within the entire cohort. To explore variation between bacterial communities, counts from distinct ASVs belonging to the same species were merged (**Supplementary File 1**). Bray-Curtis distances (β diversity) between all samples were calculated and between-sample variation was examined by performing principal coordinate analysis (PCoA) in phyloseq (McMurdie and Holmes, 2013), then plotting the ordinations in R using ggplot2 (Wickham, 2016) from the tidyverse package (Wickham et al., 2019).

Raw sequence read files for genital tract 16S rRNA-gene profiling are available in the NCBI Sequence Read Archive (SRA) under BioProject PRJNA738803 (all 29 BioSamples in the BioProject), plus BioSample SAMN19246318 from BioProject PRJNA730929. Custom R code with associated data files sufficient to reproduce the 16S-based microbiota analysis is available as a compressed supplementary file (**Supplementary File 2**).

### Cytobrush Processing, RNA Sequencing, and RNA-Seq Analysis

Cells were gently dislodged from cytobrushes without use of enzymatic digestion, and samples were processed and sorted fresh on the day of collection. We used fluorescence-activated cell sorting to isolate four specific live (Invitrogen blue viability dye) cell populations from the cytobrush samples on a BD FACSAria Fusion using BD FACSDiva software: antigen presenting cells (APCs; CD19- (clone H1B19, BD), CD45+ (H130, BD), CD66b- (G10F5, Biolegend), HLADR+ (G46-6, BD), CD3- (UCHT1, BD), and CD11c+ (B-ly6, BD) and/or CD14+ (M5E2, BD)), CD4+ T cells (CD19-, CD45+, CD66b-, CD3+, CD4+ (SK3, BD)), neutrophils (CD19-, CD45+, CD66b+), and epithelial cells (CD19-, CD45-, large by FSC-A, EpCAM+ (94C, Biolegend)) (See **Appendix 1** for sorting strategy and **Appendix 3** for cell counts). Sorted cell populations were stored in Trizol Reagent at -80C. We then performed bulk RNA-seq on each sorted cell population from each of the 30 participants, totaling 117 samples across the four cell types. RNA was extracted using a Chloroform method and quality was assessed by TapeStation. Bulk RNA library prep was performed on these RNA samples using the SmartSeq2 protocol (Picelli et al., 2014). Size and concentration of the libraries were assessed with TapeStation and Qubit.

Pooled cDNA was sequenced three times on an Illumina NexSeq using paired-end sequencing with a 75-cycle kit to achieve adequate sequencing depth. We used fastqc (0.11.5) and multiqc (1.5) for initial quality control to flag samples with poor sequencing quality (Ewels et al., 2016). We then used STAR (2.5.2b) for alignment and mapping, eliminating samples with less than 20% uniquely mapped reads (Dobin et al., 2013). HTSeq (0.9.1) was used to count the number of reads per gene (Anders et al., 2015). All samples, across all three sequencing runs, were visualized using PCA to ensure there was no data skewing attributable to any run. There was no evidence of batch effect across sequencing runs (data not shown), so the data from the three runs was merged for analysis. Next, the mitochondrial transcriptional content of each sample was assessed using EnsDb.Hsapiens.v75 in R. Because the percentage of mitochondrial transcript content was significantly different between cell types, different mitochondrial content cutoffs were used in different cell types to filter out samples with a high proportion of dead or dying cells. For epithelial cells, which had significantly higher percentage of mitochondrial transcript, we used a cutoff of <50%. For all other cell types, we used a cutoff of <30% of mitochondrial transcript as a cutoff for samples to be included in downstream analysis. The gene count table was filtered to retain only genes with ≥0.5 counts per million (CPM) in at least two samples. Genes that were not approved in HGNC were also excluded. After performing all QC filtering, the transcriptome of 107 (out of 117) samples was analyzed across 18248 genes.

Differential expression analysis was conducted in R using DESeq2 version 1.30.0 (Love et al., 2014). A ranked gene list was compiled from this DESeq2 analysis and analyzed using GSEA v4.1.0 (Mootha et al., 2003; Subramanian et al., 2005). The ranked gene list was assessed for enriched gene sets using the hallmark gene list version 7.4 (Liberzon et al., 2015) and gene ontology (GO) biological processes version 7.4 (Ashburner et al., 2000; Gene Ontology, 2021). An FDR-adjusted p-value of 0.1 was used as the statistical significance cutoff. R scripts for host RNAseq analysis are available in **Supplementary File 3**.

## Results

### Microbiome Composition Across Participants

**Figure 1:**
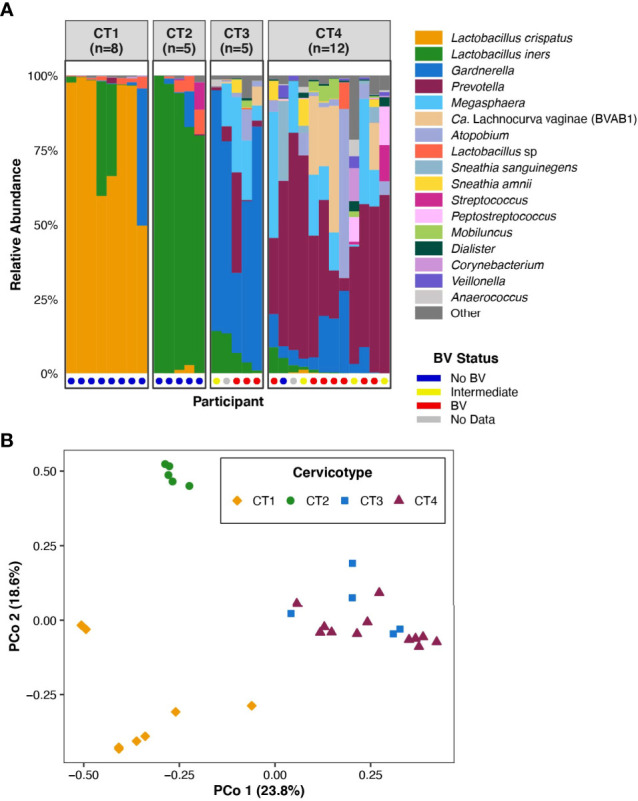
FGT microbiota of the 30 participants included in the analysis. **(A)** Bar plot representation of the microbial taxa present in the FGT in all participants, grouped by CT. Nugent score was also calculated for 28 of the 30 participants and is represented at the base of the bar plot. **(B)** Principal coordinates analysis of FGT microbial taxa from the 30 participants.

All samples and data were collected from participants enrolled in the FRESH (Females Rising through Education, Support, and Health) study in Umlazi, South Africa, who were between the ages of 18 and 23 years at the time of study enrollment and were HIV-uninfected, non-pregnant, and did not have sexually transmitted infections (STIs) at the time of sampling. We selected samples from a subset of 30 participants for paired transcriptome-microbiome analysis to ensure distribution of participants across the four vaginal microbiota community types as determined by bacterial 16S rRNA gene sequencing (**Figure 1** and **Table 1**). We have previously established a classification system that divides the FGT microbial communities into four cervicotypes (CTs) (Gosmann et al., 2017; Munoz et al., 2021). CT1 is dominated by *Lactobacillus crispatus* and CT2 is dominated by *Lactobacillus iners*. CT3 and CT4 both have higher diversity. In CT3, *Gardnerella* is the most abundant taxon, while CT4 is dominated by other taxa, typically with a significant prevalence of *Prevotella* species (**Figure 1**). Prior work has shown that women with CT3 and CT4 communities exhibit higher levels of FGT inflammation, as measured by both inflammatory cytokines in cervicovaginal lavage and by an increased frequency of inflammatory cells, including HIV target cells (activated CCR5+ CD4+ T cells), in cervical cytobrush samples (Anahtar et al., 2015). CT3 and CT4 are also associated with an increased risk of acquiring HIV (Gosmann et al., 2017).

The 30 participants examined in this analysis included eight in CT1, five in CT2, five in CT3, and twelve in CT4 (**Figure 1A**). We performed principal coordinates analysis (PCoA) based on Bray-Curtis distances to investigate the range of variation between participant vaginal microbial communities. The first two PCoA axes represented 42.4% of the total variance in the population (**Figure 1B**). Axis 1 fully segregated *Lactobacillus*-dominant (CT1 and CT2) communities from non-*Lactobacillus*-dominant (CT3 and CT4) communities, while Axis 2 fully segregated CT1 from CT2. The assignment of the four CTs (**Figure 1B**) was consistent with previous published work that studied larger sample sizes (Anahtar et al., 2015; Gosmann et al., 2017). The clear separation of FGT microbial communities based on *Lactobacillus* dominance or depletion (Axis 1, **Figure 1B**) highlighted the relevance of investigating host transcriptional differences between CT1/2 and CT3/4.

### Demographic Characteristics Across Cervicotypes

We divided the cohort based on FGT *Lactobacillus* dominance, comparing *Lactobacillus* dominant (CT1/2) to *Lactobacillus* deficient (CT3/4) communities. No factors related to sexual practices differed between groups, including the number of days since last having sex, the number of sexual encounters over the previous 30 days, and the number of sexual partners over the past 30 days (**Table 1**). There was no significant difference in reported use of condoms, vaginal drying agents, or contraceptives; however, there was a non-significant trend toward increased use of the progestin-based long-acting reversible contraceptives (LARCs) DMPA, Nuristerate, and Implanon, in the CT1/2 group. The only significant difference was in days since last reported menstrual period (LMP) (p=0.009): women with CT1/2 had a median of 45 days elapsed since LMP, longer than a regular menstrual cycle and likely reflecting the amenorrhea associated with progestin-only contraceptive methods. Women with CT3/4 had a median of 16 days since LMP, and a range within that of regular menstrual cycles. No sampling occurred during menstruation.

### Antigen Presenting Cells Exhibit a Strong Inflammatory Signature in the Non-*Lactobacillus*-Dominant FGT Microbiome

We sorted four cell lineages from each of participant: epithelial cells, APCs, CD4+ T cells, and neutrophils (**Appendix 1**, **Appendix 2**). These populations were selected based on their role in genital immune surveillance, barrier function, and HIV acquisition. We then performed bulk-RNA sequencing on each sorted cell population from each participant. In unsupervised clustering, the CTs did not cluster together (data not shown). Although we knew we were underpowered in this analysis, we proceeded with a hypothesis-generating comparison between *Lactobacillus*-dominant (CT1/2) and *Lactobacillus*-deficient (CT3/4) CTs. There were no significant differences in cell count per sample between CT groups for any cell type (**Appendix 3**) that might impact the differential gene expression analysis.

**Figure 2:**
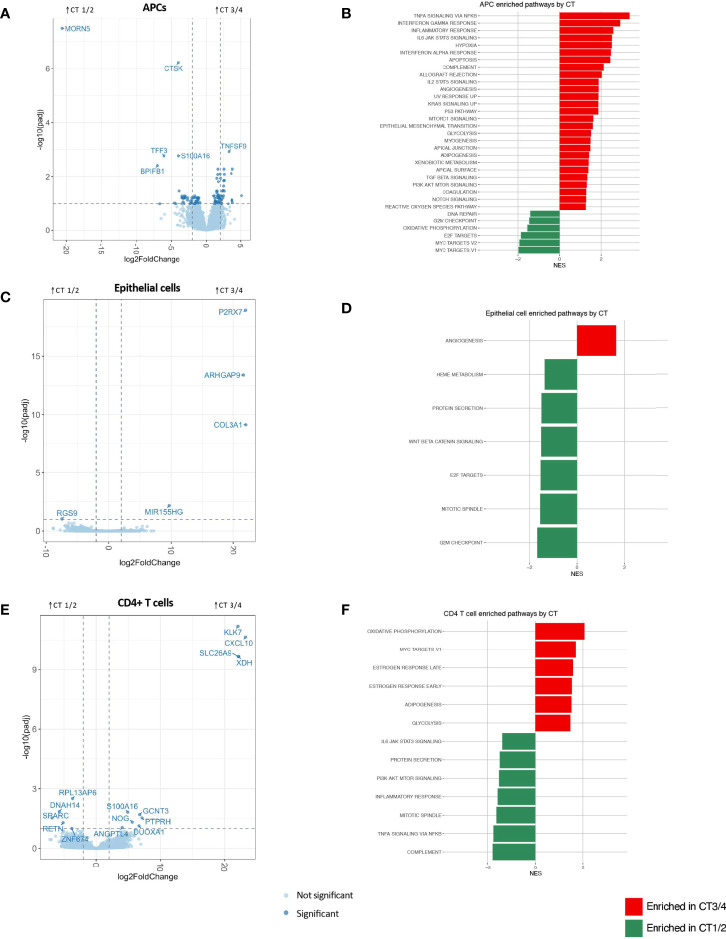
APC **(A, B)**, epithelial cell **(C, D)** and CD4+ T cell **(E, F)** differential gene expression between CTs 3/4 and 1/2 visualized through volcano plots **(A, C, E)** and significantly enriched gene sets by GSEA using Hallmark gene sets (at FDR-corrected q value of 0.1) based on Hallmark gene lists **(B, D, F)**.

APCs demonstrated the greatest number of significantly differentially expressed genes (n=111) and the most inflammatory genes and pathways associated with non-*Lactobacillus*-dominant CTs (**Figure 2A** and **Appendix 4**; **Appendix 5**). The APC genes most strongly associated with CT3/4 include those involved in the interferon response (*e.g.*, *IFIT2*, *IFIT3*, *IFIT5*, *OASL*), class I major histocompatibility complex (*e.g.*, *HLA-S*, *HLA-G*, *HLA-H*, *HLA-L*), class II major histocompatibility complex (e.g., *HLA-DRB5*), genes related to innate immunity (e.g., *MEFV*, *PELI1*, *PTGS2*, *IL1RN*), and genes promoting T-cell mediated adaptive immunity (e.g., *TNFSF9*). Genes related to regulation of neoplastic cell growth were also upregulated in the CT3/4 group (e.g., *MYCL*, *DDIT4*, *PLAC8*, and *PMAIP1*); the genes upregulated in CT3/4 predominantly suppress cell growth and/or induce apoptosis. Hierarchical clustering of samples based on genes with significant differences in expression nearly completely segregated CT1/2 from CT3/4 samples (**Appendix 4A**).

In accordance with these observations, pathways identified as significantly enriched through gene set enrichment analysis (GSEA) using hallmark gene sets in CT3/4 included multiple inflammatory pathways (e.g., TNFα signaling *via* NFkB, IFNγ response, inflammatory response, interferon alpha response, complement, allograft rejection, IL2 STAT5 signaling, and IL6 JAK STAT3 signaling) (**Figure 2B**). Pathways related to neoplastic suppression were also highlighted in this group (e.g., apoptosis, p53 pathway), as well as pro-proliferative pathways (KRAS signaling up, MTORC1 signaling, PI3K AKT MTOR signaling). Conversely, genes significantly enriched in CT1/2 included innate immune response genes (*BPIFB1*, *PELI3*), fractalkine (*CX3CL1*), genes related to mucosal epithelial integrity (*TFF3*, *CLDN10*, *MUC5B*, *LAMB1*, *RIPK4*), genes related to neoplastic invasion (*CTSK*, *KLK11*, *UCA1*), and few related to tumor suppression (S100A14). GSEA pathway analysis also highlighted proliferative signatures, such as progress through cell cycle checkpoints and Myc signaling, in CT1/2 as compared to CT3/4 (**Figure 2B**). Additional GSEA analysis performed with the gene ontology (GO) biological processes gene sets further supported APCs as drivers of dysbiosis-associated FGT inflammation, with 607 pathways upregulated in CT3/4, many of which were related to inflammatory signaling (**Appendix 5A**).

Epithelial cells represent another possible mediator of FGT inflammation. These cells exhibited very few differentially expressed genes based on microbiota composition (**Figure 2C**). No GSEA hallmark-derived gene sets appeared to have any relevance for the inflammatory signal seen grossly in the setting of CT3/4 (**Figure 2D**). A few inflammatory pathways were significantly upregulated in CT3/4 based on GO terms, but they were more related to innate inflammation, such as response to fungus, eosinophil migration, monocyte chemotaxis, and granulocyte chemotaxis, and all had relatively low normalized enrichment scores (NES 1.96-2.36) (**Appendix 5B**).

CD4+ T cells are thought to be a final common pathway of inflammation that increases risk of HIV acquisition (Haase, 2005; Haase, 2010). In this analysis, CD4+ T cells had the second greatest number of significantly differentially expressed genes after APCs (15) (**Figure 2E**). However, hierarchical clustering of samples based on differentially expressed genes was unable to segregate samples from different CTs (**Appendix 4C**), unlike the clustering observed for APCs (**Appendix 4A**). Furthermore, although a non-*Lactobacillus*-dominant FGT microbiome is known to be associated with inflammation, inflammatory pathways were surprisingly upregulated in CD4+ T cells from women in CT1/2 as compared to CT3/4 (**Figure 2F**). GO term analysis also showed some inflammatory pathways in CT1/2, such as TLR2 signaling and myeloid leukocyte mediated immunity (**Appendix 5C**), possibly suggesting a host-mediated role in curating the FGT microbiome away from BV-associated bacteria (Mares et al., 2008). The most strongly upregulated pathways in CT3/4 were related to enhanced translation capabilities (e.g. cotranslational targeting to membrane (NES 2.79), establishment of protein localization to ER (NES 2.46), translation initiation (NES 2.28)), which might relate to a CD4+ T cell activation state previously observed with dysbiosis (Anahtar et al., 2015).

Neutrophils showed no significantly differentially expressed genes between CT groups (**Appendix 4D**). The hallmark gene set “TNFα signaling *via* NFkB” was significantly enriched in CT3/4, but IFNα response was significantly enriched in CT1/2 (**Appendix 4E**), and both had relatively low normalized enrichment scores. GO term analysis similarly showed a small number of inflammatory pathways upregulated in CT3/4 (**Appendix 5D**).

Overall, these results indicate a strong inflammatory signal through APCs in the setting of *Lactobacillus*-depleted vaginal microbial communities. While other cell types may contribute to the inflammatory milieu, the prominent influence of APCs in helping to orchestrate a dysbiosis-associated inflammatory milieu is clear.

### DMPA Effects on Gene Expression

In order to explore whether hormonal effects also contribute to the transcriptional differences we observed between women with different vaginal microbiota, we compared samples from 7 women using DMPA (i.e., a high-progestin, low-estrogen state) with those from 6 women in the follicular phase of the menstrual cycle (i.e., a low-progestin, high-estrogen state; **Appendix 6**). Each hormone group was split relatively evenly by CT, resulting in 2-4 participants from each hormonal state in each CT group (**Appendix 6**). No demographic variables examined were significantly different between groups (**Appendix 6**). Due to the small sample size of an integrated analysis, we did not explicitly address the modifying effect of hormones on microbiota-associated inflammation.

**Figure 3:**
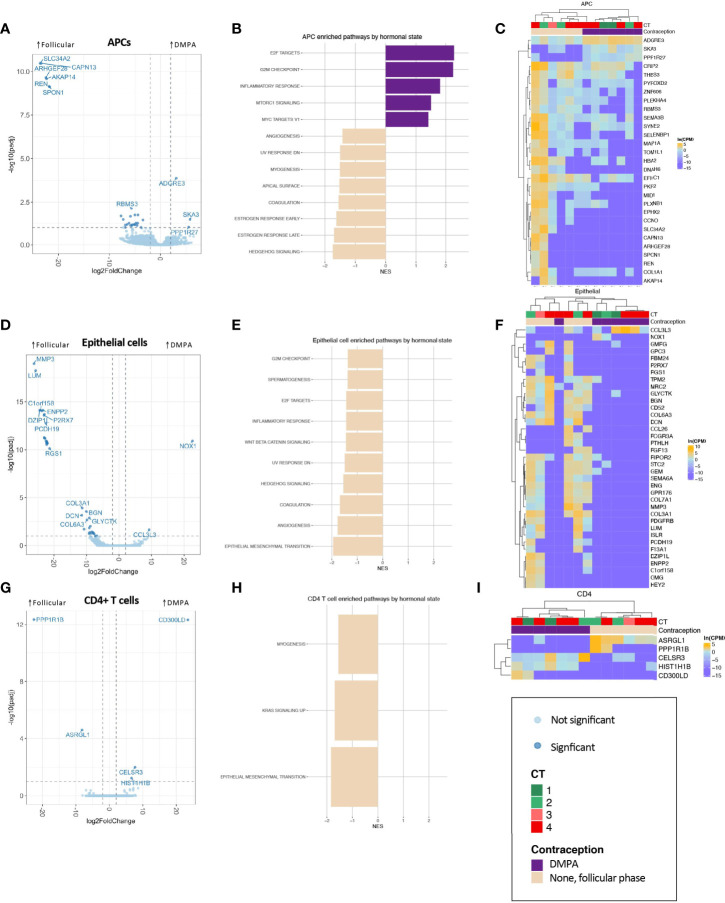
Association of DMPA with FGT APC **(A–C)**, epithelial cell **(D–F)**, and CD4+ T cell **(G–I)** transcriptional landscape. **(A, D, G)** Volcano plots showing differentially expressed genes, with significantly differentially expressed genes (BH-corrected p-value of 0.1) shown in darker points. **(B, E, H)** Significantly enriched gene sets through GSEA based on the Hallmark gene set with an FDR-corrected q-value <0.1. **(C, F, I)** Significantly differentially expressed genes are shown in heatmaps, with clustering based on Spearman distances.

Numerous transcriptional differences were observed across all cell types by hormonal state (**Figure 3** and **Appendix 8**). In APCs, epithelial cells, and CD4+ T cells, the pattern of differential gene expression nearly completely segregated women in the follicular phase from DMPA users (**Figures 3C, F, I**), suggesting a true and clear difference in transcriptional programs between groups.

In APCs, the hallmark gene set “inflammatory response” was significantly enriched (**Figure 3B**). In epithelial cells, one of the most highly differentially expressed gene across DMPA users was *CCL3L3*, which is a chemotactic cytokine for CCR5+ CD4+ T cells (**Figures 3D, F**). GO term analysis highlighted epithelial barrier differences in DMPA users (cornification, keratinization), while decreased leukocyte mediated immunity was observed in DMPA users, though with a low NES (**Appendix 9B**). Only five genes were significantly differentially expressed in CD4+ T cells (**Figures 3G, I**), but these genes were able, again, to perfectly segregate samples by hormone state.

These results demonstrate that DMPA is associated with significant changes in cellular pathways within FGT mucosal cell populations. Previous observations regarding DMPA-associated CD4+ T cell infiltrates may be partially explained by inflammation in APCs, chemotactic cytokines secreted by epithelial cells, and/or reduced epithelial barrier integrity.

## Discussion

Assessing differential gene expression characterizing cellular lineages known to be key in maintaining health of the FGT allowed us to more clearly define the transcriptional programs that underlie FGT mucosal inflammation in the context of a disrupted microbiome. Non-*Lactobacillus*-dominant vaginal microbial communities are associated with inflammation, but it has remained unclear which cell types and pathways modulate this response. Our analysis pointed strongly to APCs as a key modulator of the microbiome-associated inflammatory response. APCs exhibited upregulation of pro-inflammatory mediators in the setting of CT3/4, with increased expression of genes and pathways involved in priming of innate immunity and adaptive immunity (including T cell activation). This is consistent with previous work that highlighted APCs’ inflammatory response when treated with bacterial products *in vitro* (Anahtar et al., 2015). APCs isolated from women with CT3/4 also upregulated genes involved in cancer suppression, which may be a reflection of the inflammatory state also upregulating antiviral immunity. Conversely, CT1/2 was associated with pathways involved in cellular checkpoint progression, suggesting a decreased antiviral state. A CT3/4 associated antiviral state likely relates to recruitment of HIV target cells, CD4+CCR5+ T cells, to the FGT mucosa, increasing susceptibility to infection (Haase, 2005; Haase, 2010). The other cell types assessed (CD4+ T cells, epithelial cells, and neutrophils) did not show a strong pro-inflammatory signature in the setting of CT3/4.

To more holistically characterize the transcriptional levers acting on the FGT mucosa, we further compared two extremes of the reproductive hormone spectrum: women in the follicular phase of the menstrual cycle (high-estrogen, low-progesterone) and women using DMPA (high-progestin, low-estrogen). We found that these hormone states were associated with a large number of significantly differentially expressed genes in all cell types. Furthermore, these differentially expressed genes were able to segregate samples by their associated hormone state almost perfectly for all cell types except neutrophils. These observations highlight the strong effect of reproductive hormones, including hormonal contraceptives, on the transcriptional landscape of the FGT. Epithelial cells in DMPA users expressed the T cell homing chemokine *CCL3L3*, suggesting a pathway by which CCR5+CD4+ T cells may traffic to the FGT in the setting of DMPA use, as has been previously described (Byrne et al., 2016; Edfeldt et al., 2020). Differences in epithelial cell cornification and keratinization may also help explain increased availability of CCR5+CD4+ T cells in the FGT, consistent with previous observations (Zalenskaya et al., 2018; Edfeldt et al., 2020; Molatlhegi et al., 2020). Due to limited sample size and distribution of hormonal contraceptive methods in our cohort, we could not adequately assess for differences in transcriptional patterns between DMPA users and women in other high-progesterone, low-estrogen states, including users of other progestin-based long-acting reversible contraceptives (i.e. injectable nuristerate or Implanon), nor of women using no hormonal contraception who were in the luteal phase of the menstrual cycle. We hypothesize that many of the patterns we observe in cervix-derived cells from DMPA users would translate to these other progesterone-high states. However, interestingly, a study comparing the transcriptional and functional effects of treating cultured vaginal epithelial cells with estradiol or progesterone versus medroxyprogesterone acetate (MPA) found that MPA preferentially inhibited cell cycle progression and epithelial barrier function *in vitro* (Woods et al., 2021). Thus, larger-scale studies comparing transcriptional patterns in DMPA users versus individuals in other progesterone-high, estrogen-low states are needed to investigate whether DMPA exerts unique effects on key mucosal cell populations *in vivo*.

Together, our findings highlight the role of APCs in microbiome-associated FGT inflammation and point to the orthogonally important role of reproductive hormones in FGT inflammation. The small sample size of this analysis prohibited statistically meaningful assessment of interactions between the microbiome and hormones on the FGT transcriptional landscape. The transcriptional differences across these groups do, however, paint a compelling picture of APC-mediated inflammation due to microbiome influence, intersecting with inflammatory signals in certain hormonal contexts, with DMPA here.

As we further explore host-microbe interactions in the FGT, this study highlights the importance of considering the complex interactions that modify the FGT mucosa. The cellular effect of hormones like DMPA appears to be broader than the inflammatory pathways associated with the microbiome, primarily in APCs. Our study, in concert with previous literature, suggests that these two variables may influence FGT inflammation in lineage specific and interconnected ways to generate a common end result of FGT mucosal inflammation with increased availability of CD4+ T cells. Understanding specific cellular mechanisms of microbial and hormone associated inflammation may lead to interventions to improve reproductive outcomes, particularly in areas where the burden of these adverse outcomes is greatest.

## Data Availability Statement

Raw sequence read files for genital tract 16S rRNA-gene profiling are available in the NCBI Sequence Read Archive (SRA) under BioProject PRJNA738803 (all 29 BioSamples in the BioProject), plus BioSample SAMN19246318 from BioProject PRJNA730929.

## Ethics Statement

The studies involving human participants were reviewed and approved by the Biomedical Research Ethics Committee of the University of KwaZulu-Natal (UKZN; Ethics Reference Number BF131/11) and the Massachusetts General Hospital Institutional Review Board (2012P001812/MGH). The patients/participants provided their written informed consent to participate in this study.

## Author Contributions

MF, SB, NM, MH, and CG performed nucleic acid extractions and 16S rRNA gene sequencing from vaginal and cervical swabs. SB performed bacterial 16S rRNA gene sequencing analysis. CG developed the cell sorting panel, NX and CG performed the fluorescence activated cell sorting, and SB and NX performed the flow cytometry analysis. MF performed RNA extractions from sorted cells and RNA-Seq library preparations, and MF and JX performed sequencing of RNA-Seq libraries. KD, MD, TG, FC, TN, NI, CG, SB, NX, MG, CM and DK contributed to clinical trial design, trial performance, and/or sample acquisition and processing efforts. EB and DK conceptualized the analysis, and EB and BH performed the analysis. SH and A-CV provided analysis guidance. EB, SB, and DK wrote the paper. All authors contributed to the article and approved the submitted version.

## Funding

This work was supported by National Institutes of Health (NIH) grants R01AI111918 and Bill and Melinda Gates Foundation grant OPP1189208 to DK and HU CFAR NIH/NAIDS 5P30AI060354-15 to SB. EB was supported by award number T32GM007753 from the National Institute of General Medical Sciences. SB was supported in part by NIH grant T32 AI007387.

## Author Disclaimer

The content is solely the responsibility of the authors and does not necessarily represent the official views of the National Institute of General Medical Sciences or the National Institutes of Health.

## Conflict of Interest

The authors declare that the research was conducted in the absence of any commercial or financial relationships that could be construed as a potential conflict of interest.

## Publisher’s Note

All claims expressed in this article are solely those of the authors and do not necessarily represent those of their affiliated organizations, or those of the publisher, the editors and the reviewers. Any product that may be evaluated in this article, or claim that may be made by its manufacturer, is not guaranteed or endorsed by the publisher.
