# Supplementary material for: Antigen Presenting Cells Link the Female Genital Tract Microbiome to Mucosal Inflammation, With Hormonal Contraception as an Additional Modulator of Inflammatory Signatures
Source: Front Cell Infect Microbiol. 2021 Sep 16;11:733619. doi: 10.3389/fcimb.2021.733619 (PMC8482842; doi:10.3389/fcimb.2021.733619)

Appendix 1. (A) Schematic of FACS sorting strategy. (B) Representative flow cytometry data from a cervical cytobrush sample.

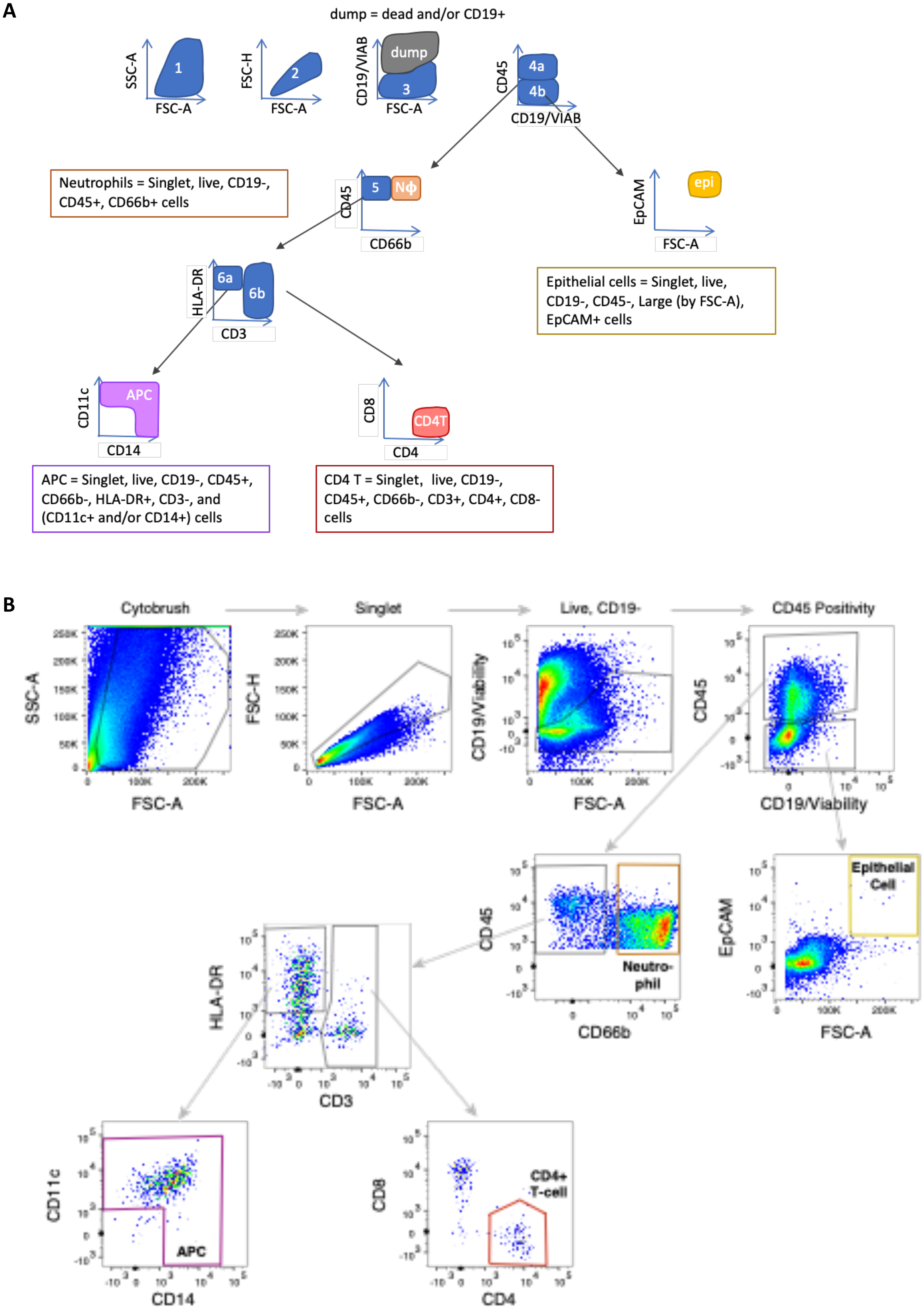

Appendix 2. The 30 participants were distributed across the cervicotypes (CTs), yielding (A) 12 or 13 samples of each cell type from women in CTs 1 and 2 and 16 or 17 samples of each cell type from women in CTs 3 and 4; (B) sample numbers decreased slightly after quality control filtering.

A

Cell type	CT 1/2	CT 3/4
APC	13	16
CD4	12	17
Epithelial cell	13	16
Neutrophil	13	17

B

Cell type	CT 1/2	CT 3/4
APC	12	15
CD4	12	15
Epithelial cell	11	14
Neutrophil	11	13

Appendix 3. Cell count per sample was not significantly different based on CT, as shown stratified by cell type (A) at time of sample selection and (B) after quality control filtering steps.

A

	CT 1/2	CT 3/4	p-value Wilcoxon (aka Mann-Whitney)
	median [IQR]		
Cell count	4080 [1238, 12032]	3397.5 [750.8, 10820.5]	0.8517
Cell count, APC	4080 [1654, 11879]	4270 [2751, 11429]	0.8799
Cell count, CD4	1168 [550, 2436]	761 [615, 4255]	0.711
Cell count, epithelial	2073 [455, 5230]	930 [169.8, 2805.8]	0.3513
Cell count, neutrophil	24006 [11293, 32121]	34202 [9649, 50000]	0.3235

B

	CT 1/2	CT 3/4	p-value Wilcoxon (aka Mann-Whitney)
	median [IQR]		
Cell count	3068.5 [988.2, 12056.5]	2983 [749, 7641]	0.884
Cell count, APC	3454 [1622, 8836]	4009 [2519, 8532]	0.8667
Cell count, CD4	1168 [550, 2436]	1670 [688.5, 4429.5]	0.4559
Cell count, epithelial	617 [318, 4806]	521.5 [159.2, 2715.8]	0.4342
Cell count, neutrophil	24246 [12032, 33331]	46637 [9649, 50000]	0.4306

Appendix 4. Further characterization of transcriptional landscape between CTs. (A-C) Heatmaps representing Spearman clustering of significantly differentially expressed genes between CTs in (A) APCs, (B) epithelial cells, and (C) CD4+ T cells. Clustering was performed using Spearman distances, except for epithelial cell samples, which could not be clustered due to low variability through the few significantly differentially expressed genes. (D-E) Neutrophil differential expression analysis showed (D) no significantly differentially expressed genes between CTs and (E) few significantly differentially enriched pathways through GSEA using Hallmark gene sets.

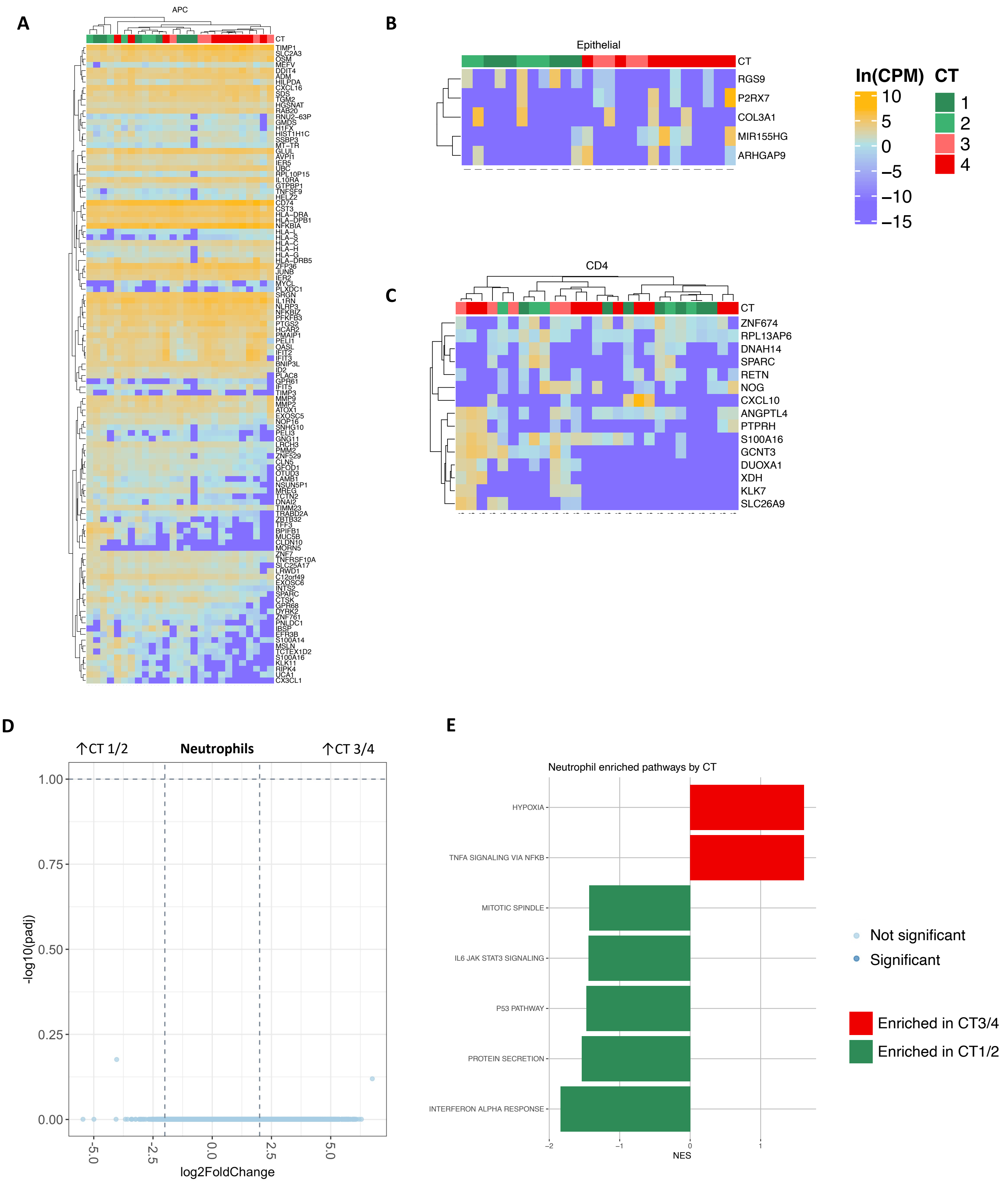

Appendix 5. GSEA pathway enrichment using the Gene Ontology (GO) biological processes gene sets for microbiome community comparison. Significantly enriched gene sets (at FDR q-value < 0.1) are shown for (A) APCs, (B) epithelial cells, (C) CD4+ T cells, (D) neutrophils.

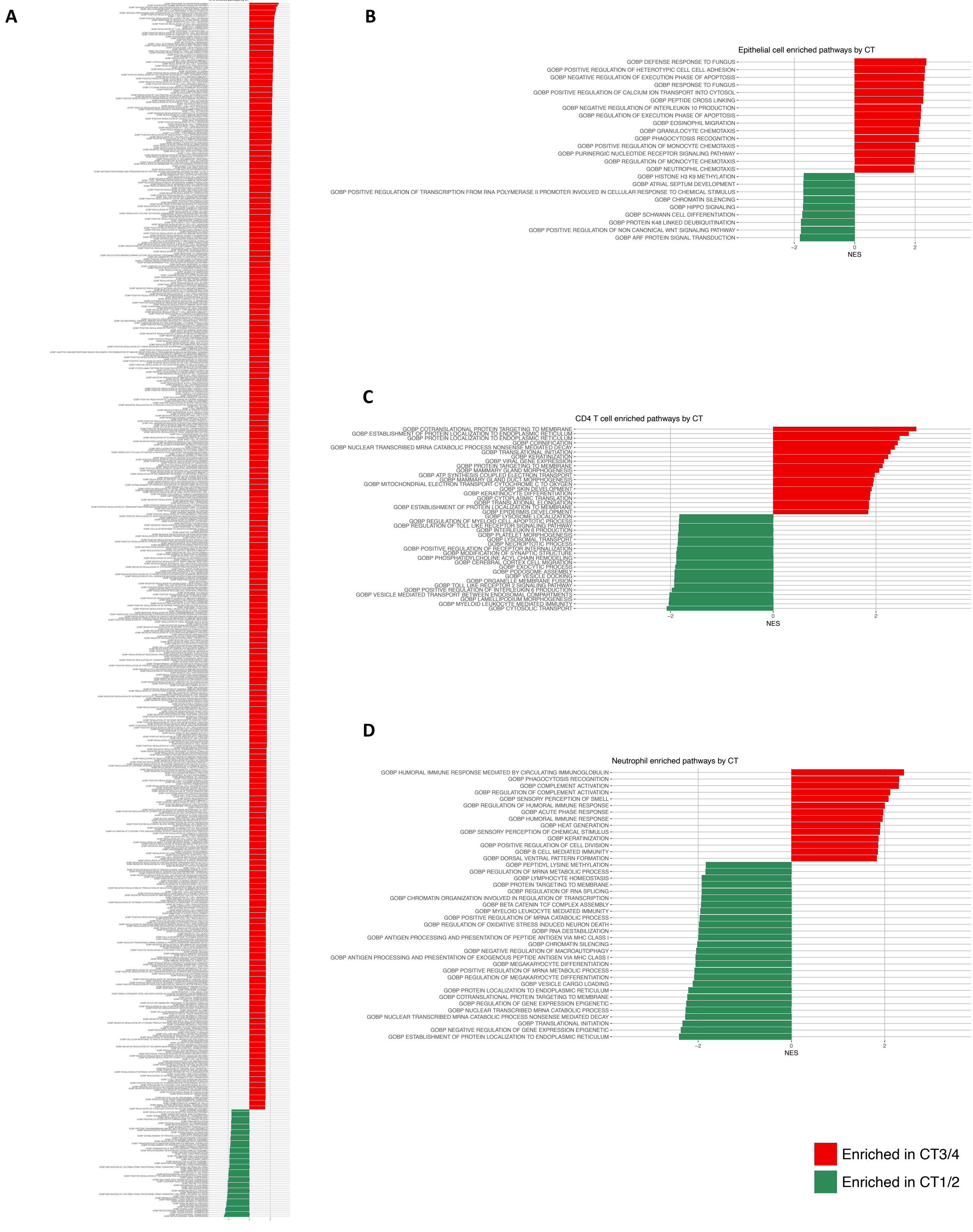

Appendix 6. Demographics of FRESH participants included in analysis (after quality control) by hormone group. All women were enrolled between the ages of 18-23 and all participants who were included in this analysis were HIV negative at the time of sampling. Data are presented as median [IQR] or number of participants, and p-values are calculated by Wilcoxon or Fisher's Exact test.

Characteristic	Follicular phase (n=6)	DMPA (n=7)	p-value
Days since last sex (median, IQR)	39.5 [12, 103]	4 [3.5, 11.5]	0.2498
Number of sexual encounters, last 30d (median, IQR)	0 [0, 3.75]	2 [1.5, 2.5]	0.3744
Number of sex partners, last 30d (median, IQR)	0 [1, 0.75]	1 [1, 1]	0.07593
Days since LMP (median, IQR)	1 [0, 2]	45 [1, 256]	0.1463
Condom use during sex			1
Always	0	1	
Sometimes	1	4	
Never	1	1	
Drying agent use			0.4615
Never	5	7	
Sometimes	1	0	
CT assignment			1
CT 1/2	2	3	
CT 3/4	4	4	

Appendix 7. The participants included women using DMPA and women in follicular phase of the menstrual cycle. After quality control filtering of samples, 5-6 samples per cell type from women in the follicular phase were compared to 6-7 samples per cell type from women using DMPA.

Cell type	Follicular phase	DMPA
**APC**	6	7
**CD4**	6	7
**Epithelial cell**	6	7
**Neutrophil**	5	6

Appendix 8. Neutrophil transcriptional differences based on hormone status. (A) Significantly differentially expressed genes are shown through darker points on a volcano plot, along with (B) significantly enriched pathways through GSEA (at FDR q-value < 0.1) using Hallmark gene sets. (C) Significantly differentially expressed genes (at BH-corrected p-value of 0.1) are shown in a heatmap with clustering by Spearman distances.

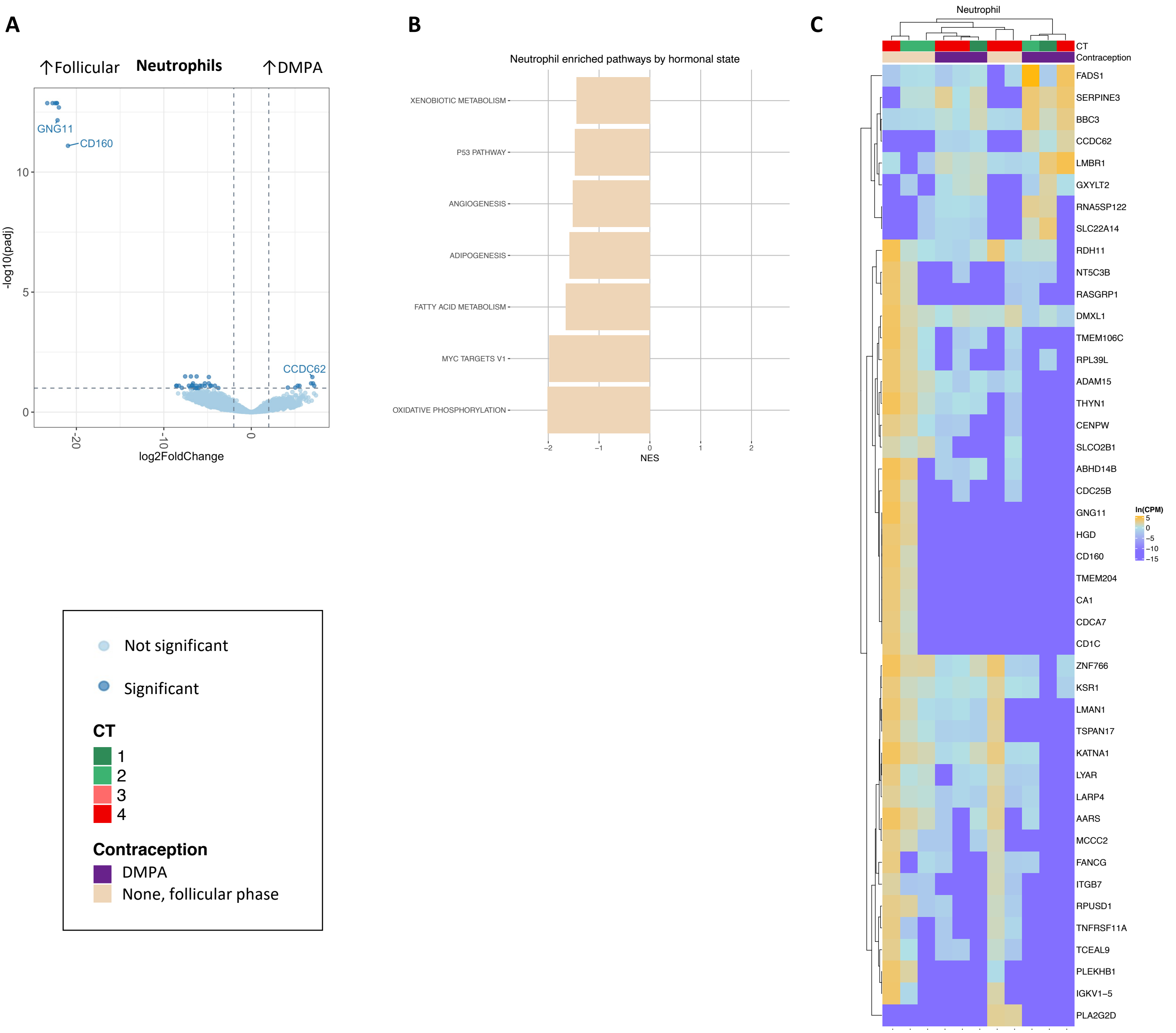

Appendix 9. Pathway enrichment using the Gene Ontology (GO) biological processes gene sets for hormonal state comparison. Significantly enriched gene sets (at FDR q-value < 0.1) are shown for (A) APCs, (B) epithelial cells, and (C) neutrophils. There were no significantly enriched pathways in either hormonal state in CD4+ T cells with this gene set.

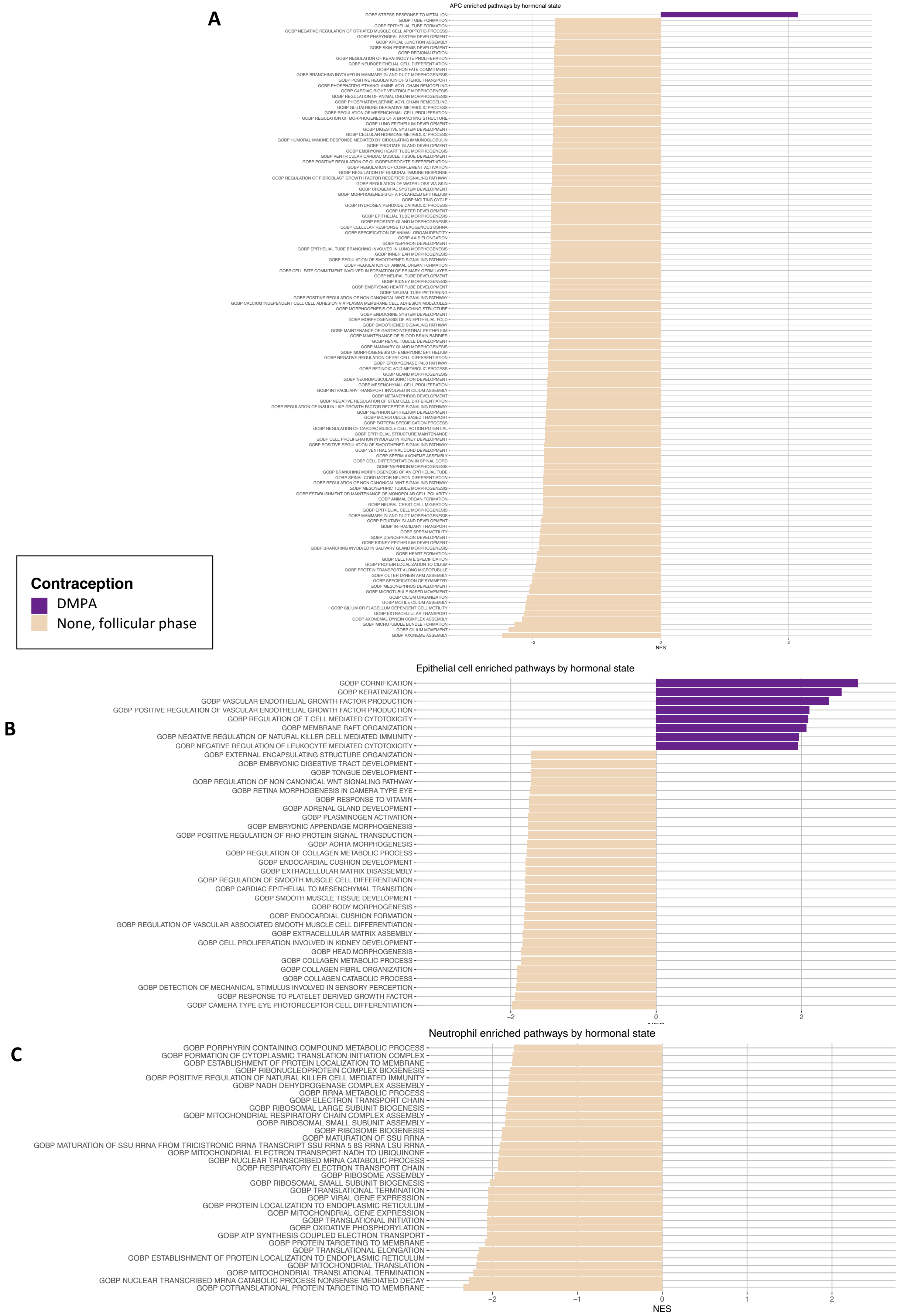

Supplement: Supplementary file 1 [file DataSheet_1.pdf]
